# Editorial: Ligands, Adaptors and Pathways of TLRs in Non-mammals

**DOI:** 10.3389/fimmu.2019.02439

**Published:** 2019-10-17

**Authors:** Jianguo Su, Xiaoqiang Yu

**Affiliations:** ^1^Department of Aquatic Animal Medicine, College of Fisheries, Huazhong Agricultural University, Wuhan, China; ^2^Laboratory for Marine Biology and Biotechnology, Pilot National Laboratory for Marine Science and Technology, Qingdao, China; ^3^School of Biological Sciences, University of Missouri, Kansas City, MO, United States

**Keywords:** Toll-like receptors, ligands, adaptors, pathways, evolution, non-mammals

Toll-like receptors (TLRs) are a pivotal family of pattern recognition receptors that are conserved in a wide variety of organisms from Porifera to mammals (Nie et al.). The typical TLRs are type I transmembrane proteins that contain three structural domains: a leucine-rich repeats (LRRs) domain, a transmembrane domain, and a cytoplasmic Toll/IL-1 receptor (TIR) domain. The LRRs domain is responsible for pathogen recognition, whereas the TIR domain interacts with signal transduction adaptors and initiates signaling. TLRs recognize microbial-associated molecular patterns (MAMPs) and damage-associated molecular patterns (DAMP), then trigger both the innate and adaptive immune systems (1, 2). Due to diverse environments and evolution, the numbers and functions of TLRs vary among different species (3). The ligands associated with the infectious agents, adaptors, and pathways of TLRs have been widely studied in mammals in spite of ambiguities and gaps in knowledge. However, they remain largely unclear in non-mammals. A better understanding of TLR pathways in non-mammals is vital to clarify immune system evolution and develop novel adjuvants and immunostimulants. The moment thus seemed appropriate for publishing a special issue on the ligands, adaptors, pathways, and evolution of TLRs in non-mammals.

Since the first TLR (Toll-1) was discovered in 1985 in *Drosophila melanogaster* embryos, functioning as the embryonic dorsal ventral polarity (4) and immune response (5), numerous TLRs have been identified in organisms from Porifera to mammals. According to the number of CF motifs (cysteine clusters at the C-terminal end of LRRs, LRRCT), TLRs can be classified into two categories: protostome-type (P-type, also known as mccTLR), and vertebrate-type (V-type, also known as sccTLR). P-type TLRs have a single cysteine cluster at LRRCT, while V-type TLRs have multiple cysteine clusters at LRRCT and sometimes even at the N-terminal end (LRRNT). P-type TLRs only exist in invertebrates; however, all of the vertebrate TLRs and some invertebrate TLRs belong to the V type (6).

The TLR repertoire in invertebrates is more abundant than that in vertebrates. This may be associated with the diversity of lifecycles, lifetimes, and environments. The number in invertebrates ranges from one (*Caenorhabditis elegans*) to hundreds of members (222 TLR-encoding genes in *Strongylocentrotus purpuratus*). TLRs in invertebrates have been identified in Porifera, Coelenterata, Platyhelminthes, Nematoda, Annelida, Mollusca, Arthropoda, Echinodermata, and Cephalochordate (amphioxus). Most invertebrate TLRs play dual roles in both developmental processes and immune responses against pathogens, but the function of vertebrate TLRs is specific to immunity (Nie et al.).

To date, at least 28 functional TLRs have been identified in vertebrates (Nie et al.). They can be divided into six subfamilies, namely, the TLR1, TLR3, TLR4, TLR5, TLR7, and TLR11 subfamilies (3). The large TLR1 subfamily, consisting of TLR1, 2, 6, 10, 14, 15, 16, 18, 25, 27, and 28, mainly recognizes lipoproteins, whereas the TLR3, 4, and 5 subfamilies recognize dsRNA, LPS (although not in fishes and amphibians), and bacterial flagellin, respectively. The TLR7 subfamily, including TLR7, 8, and 9, recognizes nucleic acid ligands. The TLR11 subfamily, containing TLR11, 12, 13, 19-23, and 26, has multiple functions, sensing proteins to nucleic acid ligands [Nie et al.; (7)]. In vertebrates, teleosts, and amphibians have the most complex TLR repertoires.

The ligands of TLRs have been widely investigated in mammals, including LPS, LTA, PG, lipoarabinomannan, flagellin, CpG-DNA, dsRNA, ssRNA, lipopeptides, envelope proteins, etc. (8, 9). However, direct evidence of recognizing and binding ligand(s) is rare in non-mammals. Fish TLR4 does not recognize LPS and negatively regulates nuclear factor-κB (NF-κB) activation (10, 11). Zebrafish have two membrane TLR5, TLR5a and TLR5b, which detect bacterial flagellin by heterodimer (12). Fish TLR9 and TLR21 recognize CpG-ODN with different CpG motifs (13, 14). Teleost-specific TLR19 recognizes dsRNA and triggers both interferon and NF-κB pathways (15).

Signal transduction in TLR pathways requires the participation of an adaptor or adaptors. There are six adaptors: myeloid differentiation primary response protein 88 (MyD88), MyD88-adaptor-like [MAL, also known as TIR domain-containing adaptor protein (TIRAP)], TIR domain-containing adaptor-inducing interferon β [TRIF, also TIR-containing adaptor molecule-1 (TICAM1)], TRIF-related adaptor molecule (TRAM, also TICAM2), sterile-α and armadillo motif-containing protein 1 (SARM1), and B-cell adapter for phosphoinositide 3-kinase (BCAP) in mammals (15–17). Amphioxus TICAM is duplicated in a basal chordate, and TICAM2 is subsequently lost in teleosts, amphibians, reptiles, and birds, and then emerges again in mammals (18); actually, evolutionary regression also exists in TLR15 (Voogdt et al.). MyD88 mediates a universal pathway for all the TLRs except TLR3 and TLR19. MAL acts as a partner for MyD88 in the TLR4-initiated MyD88-dependent pathway. TRIF is specifically involved in TLR3 and TLR19 signaling and, when coupled to TICAM2, it can also be recruited by TLR4, leading to the production of type I interferon. SARM1 and BCAP are negative regulators in TLR signaling (19, 20).

To date, TLR pathways are divided into two types: the MyD88-dependent pathway and the TRIF-dependent pathway (Zhao et al.). The MyD88-dependent response is utilized by almost all the TLRs, with the exception of TLR3 and TLR19 (15) and finally results in NF-κB transcription for inflammatory responses. The TRIF-dependent pathway is considered to be specific for just a few TLRs. The TLR recognizes a ligand, followed by the recruitment of TRIF. TRIF triggers TANK-binding kinase 1 (TBK1), phosphorylates IRF3, and activates NF-κB, activating protein-1 (AP-1) and interferon (Hu et al.; Zhao et al.). A co-evolutionary relationship has existed between TLRs and the MyD88-NF-κB pathway ever since the first emergence of rudimentary TLR in Porifera. TRIF-mediated TLR signaling appeared much later. Although TRIF ortholog has evolved in the basal chordate amphioxus, it does not induce the production of type I interferon (18). Actually, interferon regulatory factor 3 (IRF3) and IRF7, the essential transcription factors for type I interferon production, have not been identified in phyla lower than the jawed cartilaginous fish. Hence, the antiviral interferon system only exists in vertebrates.

In general, TLR pathways appear essentially conserved in evolution, especially in vertebrates. However, various members and functions in different species should not be neglected. Related research has been relatively rare in non-mammalian species. Hence, further systematic and integrated studies are expected to construct the TLR network in the future.

## Author Contributions

JS wrote the manuscript. XY edited and contributed to the organization of the editorial article.

### Conflict of Interest

The authors declare that the research was conducted in the absence of any commercial or financial relationships that could be construed as a potential conflict of interest.
